# Response of Tomato Genotypes under Different High Temperatures in Field and Greenhouse Conditions

**DOI:** 10.3390/plants10030449

**Published:** 2021-02-27

**Authors:** Sophoanrith Ro, Leangsrun Chea, Sreymey Ngoun, Zachary P. Stewart, Siranet Roeurn, Penghieng Theam, Sathya Lim, Rathana Sor, Meas Kosal, Malean Roeun, Kim Sreang Dy, P. V. Vara Prasad

**Affiliations:** 1 Faculty of Agronomy, Royal University of Agriculture, Dangkor District, Phnom Penh P.O. Box 2696, Cambodia; cleangsrun@rua.edu.kh (L.C.); ngounsreymey@rua.edu.kh (S.N.); siranet@gmail.com (S.R.); theam_penghieng@rua.edu.kh (P.T.); limsathya24@gmail.com (S.L.); srathana@rua.edu.kh (R.S.); kosalmeas2015@gmail.com (M.K.); roeunmalean25@gmail.com (M.R.); dykimsreang168@gmail.com (K.S.D.); 2 Feed the Future Innovation Lab for Collaborative Research on Sustainable Intensification, Kansas State University, Manhattan, KS 66506, USA; zastewart@usaid.gov (Z.P.S.); vara@ksu.edu (P.V.V.P.); 3 Department of Agronomy, Kansas State University, Manhattan, KS 66506, USA

**Keywords:** fruit yield, growing conditions, heat stress, high temperatures, tomato

## Abstract

Heat stress is one of the production constraints for tomato (*Solanum lycopersicum* L.) due to unfavorable, above optimum temperatures. This research was undertaken to evaluate growth and fruit yield of tomato genotypes under three contrasting growing conditions (i.e., optimal temperature in field-, high temperature in field- and high temperature in greenhouse conditions) to determine their relative heat tolerance. Eleven tomato genotypes, including two local check varieties, were evaluated, and data on growth and yield were measured and analyzed. The interactions between the genotypes and growing conditions for all yield traits were significant. In general, the performance of tomato under optimal temperature field conditions was better than under high temperature field- and greenhouse conditions. Genotypes CLN1621L, CLN2026D, CLN3212C, and KK1 had consistently greater fruit yield per plant in all growing conditions. Although the local genotype, Neang Tamm, had lower yield under optimal conditions, it performed moderately well under high temperature field- and high temperature greenhouse conditions, and yield decrease under high temperature condition was minimal. Genotype CLN1621L had stable fruit setting compared to other genotypes under high temperature conditions. Since fruit setting and yield are important traits for heat tolerance, genotypes CLN1621L and Neang Tamm are potential candidates for breeding programs focused on improved yield and heat stress tolerance.

## 1. Introduction

The Solanaceae family consists of more than 3000 species [1], among which tomato (*Solanum lycopersicum* L.) is one of the most important vegetables in the world [2]. In 2018, tomato was cultivated in 4.8 million ha worldwide and almost 80,000 ha in Southeast Asia [3], making tomato the most important vegetable crop in the region. Tomatoes have significant market potential and are considered a high value vegetable in Cambodia. Due to rapid population growth, it is necessary to improve vegetable production to meet domestic demand. Tomatoes in Cambodia’s markets are mostly imported from Vietnam [4]. 

Although this crop is grown in a wide range of climatic conditions from temperate to hot and humid tropics [5], its production is threatened and impacted by abiotic constraints, which limits productivity [6,7]. Type and occurrence of abiotic stresses often vary according to growing seasons. Among those abiotic stresses, heat stressis common and results in lower crop productivity. 

In Cambodia, the maximum average temperature is 37 °C, especially near the end of the dry season from March–April. Furthermore, Cambodia’s annual temperature has increased by 0.8 °C since 1960 with a rate of approximately 0.18 °C per decade due to climate change, and is projected to increase by 0.7–2.7 °C by 2060 and by 1.4–4.3 °C by 2090 [8]. The yield of tomatoes is known to be sensitive to high temperatures [9,10]. When temperature exceeds 35 °C, fruit setting declines [11]. Such extreme temperature occurs during the dry season in Cambodia, causing a low or an absent local production of tomato during this period. Thus, local crop production during the dry season is not sufficient to fulfill the local demand. It is necessary to improve heat tolerance of tomato in order to increase the local production. Zhou et al. [12] reported that the sensitivity to high temperature varies with genotypes. This finding implies a possibility to identify high temperature tolerant genotypes, which provides options to produce and manage tomato crops during dry season and in achieving high yield and fruit quality.

In Cambodia, tomatoes are produced under both open-field and greenhouse-conditions. Greenhouse production can ensure year-round production [13]. In recent years, greenhouse-based tomato production in Cambodia has been increasingly popular, as it is believed to minimize the risk of crop failure caused by pests, diseases, and abiotic stresses. However, production in such controlled environments requires resources, which increase production costs, especially when a micro-climate-controlled system is included. Thus, most crop production in polyethylene-roofed greenhouses of small- and medium-holder farmers do not have micro-climate controlling systems, which generally increases internal temperature, causes heat stress, and decreases yields. The responses of genotypes to high temperature under open field conditions are not clearly understood or quantified since it involves confounding effects from other environmental factors [14]. Thus, this research was conducted in both field and greenhouse conditions to fully evaluate tomato genotypes and their performance in various conditions. Ideal genotypes should not only have high yield potential, but also yield consistency and stability under varying environmental conditions [15]. Thus, the objective of this study was to evaluate key physiological traits and fruit yield of different tomato genotypes grown under three contrasting environmental conditions (optimum temperature in field-, high temperature in field-, and high temperature in greenhouse conditions) to determine their relative heat tolerance. 

## 2. Results

The analysis of variance revealed significant effects for genotypes and growing conditions and their interactions on all recorded traits (Table 1). There were significant differences (*p* < 0.01) among the responses of tomato genotypes to different environmental conditions (Table 1, Table 2, Table 3 and Table 4). Across all the measured parameters, all the genotypes performed better in optimal temperature conditions than in high temperature field and greenhouse conditions (Table 2, Table 3 and Table 4).

### 2.1. Fruit Size Traits

Within conditions, there was a significant difference in all fruit size traits (Table 2), and significant interaction between genotypes and environmental conditions (Table 1). In general, both fruit length and diameter of tomato genotypes was reduced under HTFC and HTGC compared to OTFC; except for genotypes CLN2498D and Neang Tamm. Overall, averaged across all genotypes fruit length decreased by 14.8% and 12.1% under HTFC and HTGC, respectively, compared to OTFC (Table 2). The corresponding decreases in fruit diameter were 16.6% and 10.6%, respectively (Table 2). Some genotypes such as CLN3736D, CLN3024A, and CLN2026D showed apparent decrease in fruit length and diameter under HTFC and HTGC. The highest fruit length and largest fruit diameter were observed for genotypes CLN3125L and local KK1, respectively, in all the three environmental conditions.

### 2.2. Fruit Yield Traits

There was a significant interaction between genotypes and environmental conditions for all fruit yield traits (Table 1). In general, the fruit setting of the genotypes decreased when grown under HTFC and HTGC compared to OTFC (Table 3). Between high temperature conditions, the genotypes produced higher fruit setting in HTGC than in HTFC. A sharp decrease in fruit setting under high temperature of either field or greenhouse condition was observed for genotypes CLN3078G and CLN2498D. Under OTFC, genotype CLN3736D had the highest fruit setting (62.59%). Whereas, genotype CLN1621L had the highest fruit setting (42.26%) under HTFC. Although both local Neang Tamm and KK1 had significantly lower fruit setting in all the three environmental conditions than imported genotypes, the high temperature conditions did not decrease fruit setting in local varieties. Irrespective of temperature conditions, fruit setting of local genotype KK1 was lower in open-field than in greenhouse condition. 

The overall trend of genotypes under OTFC had more plant biomass than high temperature of both conditions (HTFC and HTGC) (Table 3). The plant biomasses under HTGC were the lowest for most genotypes except for Neang Tamm, which had similar yield under different environmental conditions. The local genotype Neang Tamm consistently had the lowest plant biomass compared to other genotypes.

Most imported genotypes produced higher yield under OTFC, while they produced lower fruit yield under two other high temperature conditions (HTFC and HTGC), for, e.g., genotypes CLN3736D, CLN3078G, and CLN3024A (Table 4). Under OTFC, most imported genotypes, except CLN3212C and CLN2498D, yielded the highest. Despite the lowest fruit setting, the local genotype KK1 produced consistently the higher fruit yield in all the conditions. Following KK1, genotypes CLN1621L and CLN3212C produced higher fruit yield compared to other genotypes under high temperature of either HTFC or HTGC. Local genotype Neang Tamm produced the lowest fruit yield under OTFC whereas it yielded on par with few of the improved genotypes under HTGC. 

**Figure 1:**
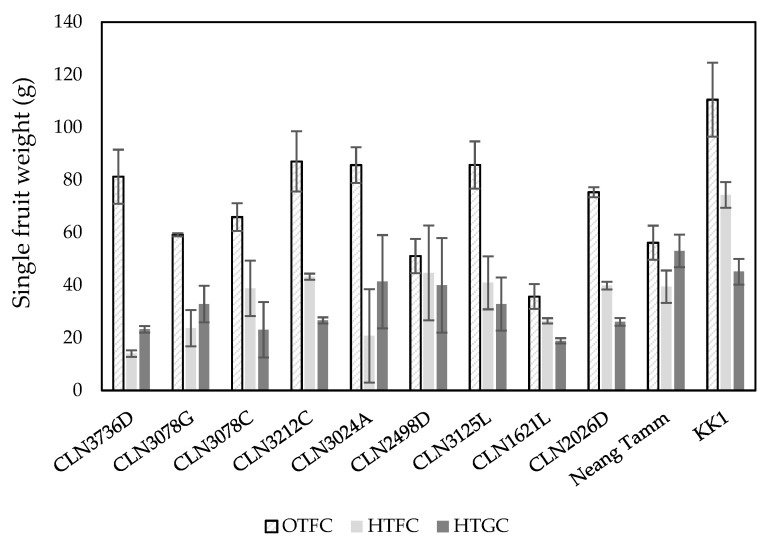
Single fruit weight of tomato genotypes under optimal temperature field condition (OTFC), high temperature field condition (HTFC), and high temperature greenhouse condition (HTGC). Value is the mean of three replications with the error bars which represent standard deviation.

Compared to OTFC, the single fruit weight (SFW) decreased significantly under high temperatures of both field and greenhouse conditions (Figure 1). Under OTFC, local genotype KK1 had the highest SFW while genotype CLN1621L was the lowest SFW. The differences of SFW between the two high temperature conditions were not consistent. Under HTFC condition, local genotype KK1 had highest SFW, while the lowest was observed for genotype CLN3736D. The SFW of both local genotypes tended to be higher. The SFW of genotype Neang Tamm was significantly higher than genotypes CLN3736D, CLN3078C, and CLN1621L.

**Figure 2:**
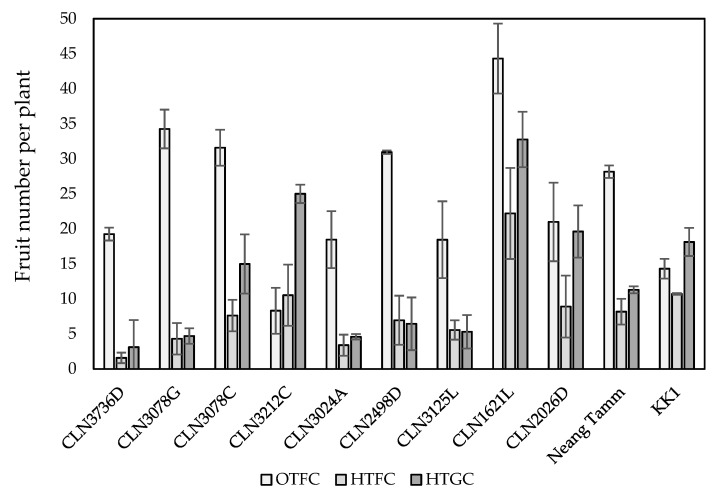
Fruit number per plant of tomato genotypes under optimal temperature field condition (OTFC), high temperature field condition (HTFC), and high temperature greenhouse condition (HTGC). Value is the mean of three replications with the error bars which represent standard deviation.

The fruit number per plant (FNPP) was higher under OTFC with exception of genotype CLN3212C and local KK1 (Figure 2). Under OTFC, genotype CLN1621L had the highest fruit numbers. Between the two high temperature conditions, the FNPP of most genotypes under HTGC was higher than HTFC. 

### 2.3. Chlorophyll Index

**Figure 3:**
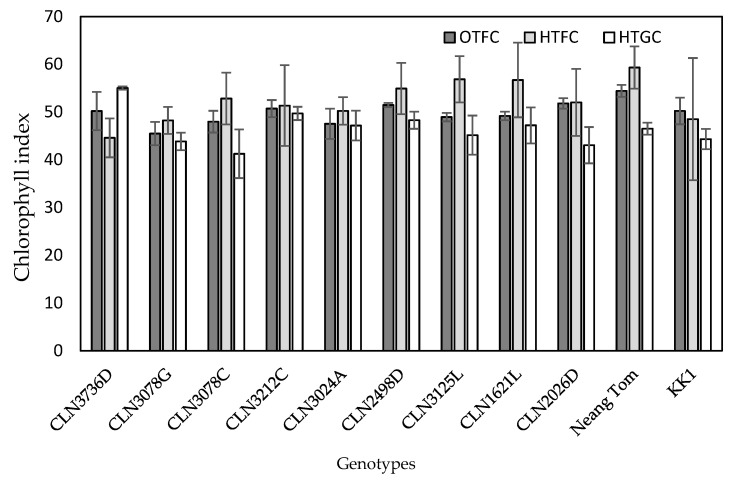
Chlorophyll index (SPAD readings) of the genotypes in both field (optimum temperature field condition (OTFC), high temperature field conditions (HTFC), and high temperature greenhouse conditions (HTGC). Value is the mean of three replications with the error bars which represent standard deviation.

Except for genotype KK1, in general the chlorophyll index (SPAD readings) was greater in both field conditions compared to greenhouse conditions (Figure 3). There was significant difference in the chlorophyll in OTFC and HTGF, but not in HTFC.

### 2.4. Yield Deviation from OTFC

Compared to OTFC, the fruit yield decreased dramatically (6.51–98.59%) under both HTFC and HTGC conditions for several genotypes, for, e.g., CLN3736D, CLN3078G, CLN3024A, and CLN3125L had the lowest fruit yield with the reduction of yield between 83.37–98.59% (Table 4). The least decrease in fruit yield under HTFC and HTGC was observed for genotypes CLN3212C (7.93–37.11%) and local Neang Tamm (6.51–49.73%). The fruit yield of KK1 was reduced approximately 50% when grown under HTFC and HTGC.

## 3. Discussion 

**Figure 4:**
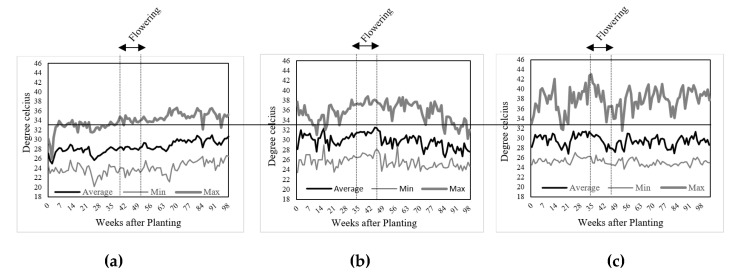
Temperature during the whole crop cycle of optimal temperature field (**a**), high temperature field (**b**), and high temperature greenhouse conditions (**c**). The solid line is a critical temperature at 33 °C for tomato production.

Tomato performance between growing conditions varied considerably. The chlorophyll index and fruit yield under high temperature of both field (HTFC) and greenhouse conditions (HTGC) were lower than in optimal temperature conditions (Table 2, Table 3 and Table 4) due to lower fruit setting. Reduced yield and lower fruit setting under high temperatures resulted in lower fruit numbers may be a result of poor pollen quality and viability, as observed in tomato [16,17] and other crops [18,19]. The yield losses due to high temperature in our study was around 70%, which was much higher than previous report by Alsamir et al. [20] with only 28% yield reduction. This difference could be due to the timing, intensity, and duration of exposure to high temperature stress during different stages of crop development. Tomatoes grown in the two conditions (HTFC and HTGC) experienced above optimum high daytime temperatures, particularly during the periods of flowering and fruit formation (Figure 4). Temperature conditions in greenhouse experienced above optimum temperatures for longer duration or throughout the season relative to the field conditions. A significant difference in the diurnal temperatures (relative different in daytime high temperature and nighttime minimum temperatures) was observed for these three conditions (Figure 4), although the mean temperatures under these conditions was similar (28, 29, and 29 °C for OTFC, HTFC, and HTGC, respectively). For yield formation or fruit set, the maximum or minimum temperature may have greater impacts than the mean temperature throughout growing season. This is due to fact that even exposure to short periods of above optimum high temperatures during the sensitive reproductive stages of crop development can significantly influence reproductive traits (particularly gamete viability) leading to significant yield losses in many crops [21] and the response depends on timing, intensity, and duration of heat stress [22,23]. 

Between the high temperature conditions, the fruit yield of some genotypes in the field conditions was lower than the greenhouse conditions in which greater maximum temperature was recorded in the present study. Similar trends for higher fruit yields per plant in the controlled environment relative to open field conditions were reported by Kanwar [24]. These results were probably due to differences in other abiotic and biotic factors rather than temperature in the field [6,7], as our results indicated that overall performance tended to be better in the greenhouse even while facing greater maximum temperatures than the high temperature field conditions. 

Chlorophyll index readings are positively correlated with leaf chlorophyll content [25,26,27]. High temperature adversely affects chlorophyll content, photosynthesis rate, and consequently plant biomass production [28]. In the present study, a decrease in chlorophyll index of most genotypes was observed for HTGC compared to OTFC (Figure 3). It could be due to the fact that very high temperature under greenhouse condition may lead to stomata closure; therefore, lower photosynthetic rate [28]. This resulted in greater dried biomass under OTFC and HTFC than under HTGC (Table 3). This was exception for genotype CLN3736D which had high chlorophyll index under HTGC. The mechanisms associated to greater chlorophyll index under HTGC for this genotype needs further investigation. This could be associated with the rate of chlorophyll breakdown or degradation which changes with leaf senescence and during fruit ripening in tomato [29]. A recent study on tomato observed that chlorophyll index was greater under heat stress [30]. The impact of heat stress on chlorophyll content of tomato can vary with genotypes, temperature, and stage of development and their interactions [12].

Most of the tested genotypes produced higher yield under OTFC; however, the yield decreased dramatically under high temperature conditions. In all conditions tested, the local genotype KK1, tended to produce the greater yield due to large fruit length and diameter rather than fruit setting, a trait considered to confer a plant tolerance to high temperature [31]. Another local genotype Neang Tamm performed moderately in terms of fruit yield under high temperature conditions in which the yield decrease was relatively low, but performance under optimal conditions was poor. The yield of genotype KK1 was followed by CLN1621L, CLN3212C, and CLN2026D under high temperature conditions. Even though the three genotypes are rated as “good” for heat tolerance [32], CLN1621L was superior under high temperature conditions as the genotype could maintain high fruit settings (Table 3) across conditions in spite of smaller fruit as indicated fruit size traits and single fruit weight (Figure 1). Dane et al. [31] reported that genotypes with small fruits and more flowers were less affected by heat stress than larger fruited genotypes. Similar results were reported by Solankey et al. [33] in that CLN1621L was among the most heat tolerant genotypes. Sangu et al. [34] reported that temperatures up to 35/28 °C day/night did not affect flower development of genotype CLN1621L and had higher fruit set as observed in our study. Fruit set and yield are important traits associated with heat tolerance and are used in tomato screening. The genotypes CLN1621L and Neang Tamm have the potential to be a donor in breeding programs to enhance heat tolerance when crossed with other existing local genotypes, and may result in overall better genotypes with greater tolerance and fruit size traits. However, better understanding of the mechanisms associated with heat tolerance in CLN621L and Neang Tamm needs further investigation. In addition, the ability of these genotypes in crossing programs, inheritance of traits such as fruit set, and hybrid performance is needed. 

Even though we characterized the growth performance and fruit yield of these tomato genotypes, there is need for further evaluation of these genotypes for other heat tolerance traits such as pollen number per flower, pollen viability, cell membrane stability, photosynthetic performance, and molecular mechanisms [35], which may identify high temperature tolerance genes for breeding heat-tolerant hybrids [9]. A combination of improved genotypes with better heat-tolerant traits and other crop management of other biotic constraints such as disease should also be taken into account to improve tomato production in Cambodia.

## 4. Materials and Methods 

### 4.1. Experimental Site

This research was conducted under field conditions at Crop Station (11°30′46″ N, 104°54′1″ E) and greenhouse environments at Royal University of Agriculture (RUA), Cambodia. Selected genotypes were grown (a) under high temperature field condition (HTFC); (b) high temperature greenhouse condition (HTGC); and (c) optimal temperature field condition (OTFC). The soil properties were analyzed at the soil laboratory of RUA and are presented in Table 5.

Although there were three different experiments, the differences in soil composition were minimal. To ensure that there was no interference in soil composition factor, the same recommended dose of fertilizer by Cambodian Agricultural Research and Development Institute (CARDI) was applied to the three experiments. 

### 4.2. Temperature 

Temperature data loggers (HOBO UX100-003; Onset Computer Corporation, Bourne, MA, USA) were installed to record temperatures at 15 min intervals throughout the three growing conditions. The HTFC research was conducted during the dry season (March to June, 2018), where crops are often exposed to high temperatures. The HTGC research was conducted during the rainy season (July to October, 2018); however, the crop was exposed to high temperatures by not installing a micro-climate controlling system. The OTFC research was carried out during optimal temperature condition (December 2018 to March 2019). The weather in Cambodia starts to cool down in November. The temperatures normally start to rise by March. Thus, the growing condition of OTFC experiment was considered as an optimum condition (Figure 4). Temperature ranged from 23.47 °C to 38.84 °C in HTFC with a mean temperature of 29.87 °C, from 23.62 °C to 42.98 °C in HTGC with the mean temperature of 29.43 °C, and from 20.13 °C to 37.65 °C with the mean temperature of 28.08 °C in OTFC (Figure 4).

### 4.3. Plant Material 

Eleven tomato genotypes (Table 6) including two local varieties, Neang Tamm from CARDI and KK1 from the Kbal Koh Vegetable Research Station (KVRS), Cambodia, were selected for the study. The two local varieties were released by these two local research institutes. The other nine tomato genotypes were obtained from the World Vegetable Center (Tainan, Taiwan). The selection of these genotypes was based on suitability to target environments fitting to the Cambodian environment (warm dry and hot dry), growth habit (determinate and semi-determinate), and relative heat tolerance (moderate to good). 

### 4.4. Experimental Layout and Management 

#### 4.4.1. Seedling Preparation 

Three seeds were sown in multi-pot trays consisting of 50% well-fined compost and 50% alluvial soils. The seedlings were thinned after one week. After 21 days, a single seedling was transplanted per hill into soil for field environment; or in pots in greenhouse environment. 

#### 4.4.2. Environment 1: Optimal Temperature Field Condition (OTFC)

In this environment, the selected genotypes were arranged randomly with three replications. The plot size was 1.4 m width by 4 m length. Sixteen tomato seedlings were planted at a spacing of 50 cm by 70 cm in each plot with two rows of crops. All plots were mulched with rice straw at a rate of 10 t·ha^−1^. The replacement of weak or dead seedlings was done within a week after planting.

Fertilizer was applied according to the recommendations of CARDI for tomato production at a rate of N: P_2_O_5_: K_2_O at 75: 30: 100 kg·ha^−1^ [36] and well-composted cattle manure at 20 t·ha^−1^. The nutrient composition of the cattle manure was total N 1.18% (Kjeldahl digestion), P_2_O_5_ 9.96% (Olsen method), exchangeable K 0.95% (Flame photometer), organic carbon 83.57% (Ignition loss). The cattle manure, all mineral P and K, and 50% of N were applied as basal. The remaining 25% N was applied at the first flower blooming and 25% after the first fruit harvest.

#### 4.4.3. Environment 2: High Temperature Field Condition (HTFC)

The plot design and mineral fertilizer application were the same as described in environment 1 with three replications. The well-compost cattle manure was obtained from the same source as environment 1. Thus, the nutrient composition was assumed to be the same. 

#### 4.4.4. Environment 3: High Temperature Greenhouse Condition (HTGC)

The greenhouse research was arranged with three replications (one plant per pot and five pots per replication). The pots were arranged with a spacing of 50 cm by 70 cm. The size of the greenhouse was 5 m by 20 m, roofed with polyethylene, and sided by anti-insect net with 0.4 mm mesh. A healthy seedling was planted in each pot filled with 18 kg of air-dried soils. The plastic pots had a height of 35 cm and diameter of 25 cm. 

For the greenhouse trial, fertilizer application rates were calculated and converted into weight per pot based on plant density. The basal fertilizers were thoroughly mixed with the soil before transferring into the pots. The remaining fertilizers were top-dressed. 

Tomatoes were allowed to self-pollinate without flower vibration. 

### 4.5. Pest Control 

Metalaxyl and mancozeb (0.6g·L^−1^) were applied at one and five weeks after planting (WAP) to control fungi-caused diseases in all environmental conditions. In addition, copper hydroxide (1 g·L^−1^) was also applied at three and six WAP.

In the field conditions, imidacloprid was used to control white fly at the dose of 5cc 25 L^−1^ at two, four, and six WAP. Abamectin was used for worm control in field conditions at the recommended dose of 5cc 25 L^−1^ during fruit formation.

### 4.6. Irrigation

In all environmental conditions, the plants were irrigated on a daily basis using a drip irrigation system. The amount of water applied was based on visual observation and if needed, the plants were irrigated up to two times per day. 

### 4.7. Chlorophyll Index Reading

A portable SPAD-502 (Minolta Camera Co., Osaka, Japan) was used to assess leaf chlorophyll index of each genotype. The calibration of the SPAD-502 meter was conducted prior to data collection. Two uppermost, fully developed leaves of five random plants per plot were measured. The measurement was carried out at four and six WAP. The reported measurements are the average of the two-measurement timing and three replications. 

### 4.8. Fruit Harvesting and Data Collection 

Five plants per plot were randomly sampled for data collection in the field environments, and all potted plants were sampled in the greenhouse environments. Tomato fruits were harvested six times at 9, 10, 11, 12, 13, and 14 weeks after planting. At each harvest, all fruits per plant were weighed and counted. 

Fruit set was calculated by dividing the number of fruits per cluster by the number of flowers per cluster. which were the means of three clusters selected from each sampled plant. Randomly, ten tomatoes of each genotype at the last harvest were sampled for fruit length and fruit diameter at each harvest using a digital caliper. 

Dry weight was collected including the mass of leaves and stems cut above the soil surface after oven-drying at 70 °C for 48 h.

### 4.9. Statistical Analysis 

All data were checked for normality of distribution, statistically analyzed by ANOVA for significance, and mean values were separated by Tukey’s HSD test at appropriate level of significance. A factorial analysis was performed for interaction between environmental conditions and genotypes. Both ANOVA and mean comparison were assessed using Statistix 8 (Version 8.0, Analytical Software, 1985–2003) for all the three experiments (environments). Values of all measured traits and standard deviation are based on three replications. 

## 5. Conclusions

The present study shows that there is variation of genotype performance under different environmental conditions (optimal and high temperatures) in field and greenhouse. Genotype KK1 consistently yielded the highest between all conditions due to larger fruit size, but it had lower fruit setting under high temperature conditions. Among the genotypes, CLN1621L had higher fruit set and yield under high temperatures. While local genotype Neang Tamm had lower yields under optimum temperature, it had higher yield under high temperatures. Therefore, both of these genotypes are potential candidates for heat-tolerance breeding programs. Future research should focus on determining specific mechanisms, their use in breeding programs, and inheritance of traits associated with heat tolerance. In addition, multi-location tests under different agro-ecological conditions will be required to determine the most suitable genotype in different regions in Cambodia. There is also need for deeper evaluation of fruit size and color market preferences. A combination of heat-tolerant traits (e.g., fruit setting and yield) and market traits (e.g., fruit shape and color) should be considered in breeding programs and for production in the high temperature conditions. Management of other biotic constraints such as disease and their interaction with heat stress also requires further investigation.

## Figures and Tables

**Table 1:** Significance, analysis of variance effects of main and interaction effects for different traits of tomato.

Source	df	Fruit					Biomass (g plant^−1^)	Chlorophyll Index (SPAD Reading)
		Length (cm)	Diameter (cm)	Setting (%)	Single Weight (g)	Yield (g plant^−1^)		
Genotypes (G)	10	**	**	**	**	**	**	*
Conditions (C)	2	**	**	**	**	**	**	**
G X C	20	**	**	**	**	**	**	*
Error	64							
Total	98							

*, ** Significant at 0.05 and 0.01 probability (p) levels respectively; df: Degrees of freedom.

**Table 2:** Fruit length and fruit diameter of tomato genotypes under optimal temperature field condition (OTFC), high temperature field condition (HTFC), and high temperature greenhouse condition (HTGC).

Genotypes	Length (mm)			Diameter (mm)		
	OTFC	HTFC	HTGC	OTFC	HTFC	HTGC
CLN3736D	51.62 c ± 3.01	31.45 e ± 5.06	39.39 ef ± 1.28	53.21 bcd ± 3.70	34.50 g ± 2.22	47.08 cd ± 1.21
CLN3078G	61.49 b ± 3.72	37.30 de ± 8.36	59.91 ab ± 3.49	46.22 de ± 1.16	35.55 fg ± 2.96	44.45 de ± 1.18
CLN3078C	60.56 b ± 2.03	51.53 bc ± 1.78	53.65 bc ± 6.68	46.85 de ± 2.84	39.99 def ± 0.76	46.40 cd ± 1.97
CLN3212C	54.50 bc ± 2.36	46.15 cd ± 1.86	50.35 bcd ± 0.45	58.64 b ± 5.36	48.03 b ± 2.04	53.31 ab ± 2.82
CLN3024A	55.97 bc ± 3.55	42.72 cde ± 5.36	40.90 def ± 0.23	56.82 bc ± 3.48	46.48 bc ± 1.40	35.98 f ± 1.03
CLN2498D	54.51 bc ± 3.13	62.22 ab ± 3.88	56.54 abc ± 4.43	46.81 de ± 0.04	46.22 bc ± 3.47	48.39 bcd ± 1.08
CLN3125L	78.76 a ± 1.82	71.01 a ± 3.05	63.36 a ± 2.80	48.83 d ± 1.31	41.37 cde ± 2.16	39.79 ef ± 1.70
CLN1621L	40.78 e ± 1.40	37.57 de ± 1.23	38.20 f ± 1.51	40.79 e ± 2.20	37.06 efg ± 1.01	36.40 f ± 0.22
CLN2026D	60.06 b ± 4.83	51.85 bc ± 2.53	48.34 cde ± 3.59	50.44 cd ± 2.55	41.07 cde ± 1.61	43.73 de ± 3.37
Neang Tamm	43.28 de ± 2.04	45.74 cd ± 0.48	47.24 cdef ± 1.47	47.86 de ± 2.93	44.12 bcd ± 1.17	51.20 abc ± 3.12
KK1	49.98 cd ± 2.20	43.05 cde ± 3.53	39.25 ef ± 3.98	65.97 a ± 4.20	53.90 a ± 3.85	55.07 a ± 1.82
Mean (condition)	55.59	47.33	48.83	51.06	42.57	45.62

Value is the mean of three replications ± standard deviation (SD); different letters in a column denote significant difference at *p* < 0.01 by Tukey HSD’s (Honestly Significant Difference) test.

**Table 3:** Fruit setting and plant biomass of tomato genotypes under optimal temperature field condition (OTFC), high temperature field condition (HTFC), and high temperature greenhouse condition (HTGC).

Genotypes	Fruit Setting (%)			Biomass (g plant^−1^)		
	OTFC	HTFC	HTGC	OTFC	HTFC	HTGC
CLN3736D	62.59 ab ± 14.81	11.79 de ± 4.55	25.20 bcde ± 8.01	242.78 ab ± 18.21	188.89 ab ± 38.49	141.42 a ± 8.96
CLN3078G	41.07 ab ± 6.73	8.25 e ± 2.71	8.83 f ± 3.25	278.62 a ± 55.23	194.44 a ± 34.69	105.12 ab ± 3.88
CLN3078C	50.21 ab ± 13.48	28.64 abc ± 4.35	23.82 bcde ± 1.75	259.85 ab ± 75.67	188.89 ab ± 50.92	138.82 ab ± 38.76
CLN3212C	51.34 ab ± 7.88	34.50 ab ± 5.66	32.03 ab ± 1.36	167.65 abc ± 17.95	161.11 abcd ± 41.94	65.923 ab ± 4.27
CLN3024A	47.48 ab ± 6.17	28.64 abc ± 3.23	33.50 ab ± 2.23	155 bc ± 16.17	153.45 abcd ± 35	124.83 ab ± 8.40
CLN2498D	53.82 ab ± 4.51	12.07 de ± 2.31	13.73 ef ± 2.08	183.33 abc ± 16.67	168.03 abc ± 27.60	142.27 a ± 10.74
CLN3125L	39.70 abc ± 10.13	30.20 abc ± 5.75	22.52 cde ± 5.99	277.78 a ± 38.49	178.60 ab ± 9.91	113.35 ab ± 15.08
CLN1621L	43.09 abc ± 2.57	40.26 a ± 1.63	39.06 a ± 6.32	183.33 abc ± 16.67	100.35 bcd ± 5.25	55.423 b ± 12.39
CLN2026D	38.65 bc ± 3.41	18.16 cde ± 2.26	29.09 abcd ± 4.08	178.88 abc ± 46.23	155.56 abcd ± 19.25	82.253 ab ± 34.51
Neang Tamm	65.29 a ± 6.59	22.53 bcd ± 7.39	31.10 abcd ± 2.08	72.22 c ± 63.10	70.22 d ± 26.23	85.270 ab ± 76.58
KK1	28.62 c ± 9.49	7.77 e ± 13.09	18.54 def ± 6.50	150.00 ab ± 16.67	83.13 cd ± 1.58	89.050 ab ± 3.12
Mean (condition)	38.71	22.09	36.20	205.97	149.33	103.98

Value is the mean of three replications ± standard deviation (SD); different letters in a column denote significant difference at *p* < 0.01 by Tukey HSD’s (Honestly Significant Difference) test.

**Table 4:** Mean fruit yield of genotypes under optimal temperature field condition (OTFC), high temperature field condition (HTFC) and high temperature greenhouse condition (HTGC), and mean fruit yield deviation from OTFC.

Genotype	Fruit Yield (g plant^−1^)			Mean	Fruit Yield Deviation from OTFC			
	OTFC	HTFC	HTGC		HTFC	% Decrease	HTGC	% Decrease
CLN3736D	1564.4 abc ± 432.84	22.06 f ± 5.56	72.33 f ± 13.14	552.93	−1542.34	−98.59	−1492.07	−95.38
CLN3078G	2027.0 ab ± 205.38	101.90 def ± 9.52	154.13 ef ± 42.63	761.01	−1925.1	−94.97	−1872.87	−92.40
CLN3078C	2080.6 a ± 373.10	295.91 cde ± 57.21	346.00 cde ± 191.76	907.50	−1784.69	−85.78	−1734.6	−83.37
CLN3212C	723.4 cd ± 35.01	454.94 bc ± 31.24	666.00 ab ± 88	614.78	−268.46	−37.11	−57.4	−7.93
CLN3024A	1580.9 ab ± 457.66	70.33 ef ± 3.33	190.13 ef ± 52.63	613.79	−1510.57	−95.55	−1390.77	−87.97
CLN2498D	1273.9 bc ± 341.99	310.66 cde ± 77.91	257.50 def ± 31.26	614.02	−963.24	−75.61	−1016.4	−79.79
CLN3125L	2179.7 a ± 129.30	227.29 cdef ± 15.57	174.50 ef ± 20.75	860.50	−1952.41	−89.57	−2005.2	−91.99
CLN1621L	1900.8 ab ± 6.34	586.99 ab ± 43.81	617.50 ab ± 89.75	1035.10	−1313.81	−69.12	−1283.3	−67.51
CLN2026D	2149.1 a ± 534.21	354.92 bcd ± 39.73	511.08 bcd ± 96.50	1005.03	−1794.18	−83.49	−1638.02	−76.22
Neang Tamm	640.7 d ± 67.78	322.09 cde ± 106. 31	599.00 abc ± 48.75	520.60	−318.61	−49.73	−41.7	−6.51
KK1	1707.7 ab ± 100.60	795.01 a ± 239.15	818.33 a ± 91.73	1107.01	−912.69	−53.44	−889.37	−52.08

Value is the mean of three replications ± standard deviation (SD); different letters in a column denote significant difference at *p* < 0.01 by Tukey HSD’s (Honestly Significant Difference) test.

**Table 5:** Soil properties of the field and greenhouse environmental conditions.

Soil Properties	OTFC	HTFC	HTGC
Soil pH (H_2_O, 1:2.5)	6.6	7.1	7.3
Soil organic matter (Walkley & Black wet composition)	0.76%	1.01%	1.01%
Total nitrogen (N) (Kjeldahl digestion)	0.04%	0.03%	0.02%
Available phosphorus (P) (Olsen method)	17.2 ppm	32.7 ppm	8.2 ppm
CEC (Ammonium acetate pH 7.0)	11.8 cmolc/kg	13.3 cmolc/kg	21.9 cmolc/kg
Sand	60.4	60.00%	41.50%
Silt	23.9	21.90%	34.90%
Clay	15.7	18.10%	23.60%
Texture (Hydrometer method-USDA)	Sandy loam	Sandy Loam	Loam

**Table 6:** Pedigree information of genotypes used in this research.

Genotypes	Growth Habit	Heat Tolerance	Source
CLN3736D	Semi-determinate	Fair	WorldVeg
CLN3078C CLN3078G	Determinate Determinate	Moderate Good	WorldVeg WorldVeg
CLN3212C	Semi-determinate	Good	WorldVeg
CLN3024A	Determinate	Moderate	WorldVeg
CLN2898D	Semi-determinate	Moderate	WorldVeg
CLN3125L	Determinate	Moderate	WorldVeg
CLN1621L	Determinate	Good	WorldVeg
CLN2026D	Determinate	Good	WorldVeg
Neang Tamm	Determinate	Good	CARDI
KK1	Determinate	Good	KVRS

## Data Availability

The data presented in this study are available on request from the corresponding author.

## Abbreviations

OTFC	Optimal Temperature Field Condition
HTFC	High Temperature Field Condition
HTGC	High Temperature Greenhouse Condition
WAP	Weeks After Planting
ANOVA	Analysis of Variance
G	Genotype
C	Condition
SD	Standard Deviation
HSD	Honest Significant Difference
p-value	Probability
SFW	Single Fruit Weight
